# Going down

**Published:** 1858-05

**Authors:** 


### GOING DOWN.

A Clergyman wrote to us, some time since, to know if it would not be better to give up preaching so as to rest his throat, and give it a chance to get well—he, meanwhile, taking the editorship of a religious newspaper. As well may a man become able to run a long distance without getting out of breath, by not running at all, as one suffering from an ordinary throat affection may expect to recover the power of his voice by the entire disuse of his vocal organs. Besides, we have very often noticed, that when clergymen, for any cause, sufficient or not, lay aside the gown, they seldom get fully into the traces again; it is infinitely easier to go down to a worldly calling, than to climb up to a higher and holier one. Entering the busy arena of life for money, has a contaminating effect on the mind of any man who has been once a clergyman; and the spot is seldom washed out. It is, at all events, a most dangerous experiment, and ought not to be lightly made. Sometimes this intermission is a providential means of purification, and a preparation for wider and larger influences; but it is not safe for any one to assume to himself such a supposition. So our voice was against the experiment; and in reply to some of our arguments, he states: "You speak the truth, as to at least some religious editors: the Lord save me from filling the office of a pious, influential blackguard. Still, I might fall into the same temptation."

